# Microfluidic investigation of the impacts of flow fluctuations on the development of *Pseudomonas putida* biofilms

**DOI:** 10.1038/s41522-023-00442-z

**Published:** 2023-10-03

**Authors:** Guanju Wei, Judy Q. Yang

**Affiliations:** 1 Saint Anthony Falls Laboratory, University of Minnesota, Minneapolis, MN 55414 USA; 2 Department of Civil, Environmental, and Geo-Engineering, University of Minnesota, Minneapolis, MN 55455 USA

**Keywords:** Biofilms, Applied microbiology

## Abstract

Biofilms play critical roles in wastewater treatment, bioremediation, and medical-device-related infections. Understanding the dynamics of biofilm formation and growth is essential for controlling and exploiting their properties. However, the majority of current studies have focused on the impact of steady flows on biofilm growth, while flow fluctuations are common in natural and engineered systems such as water pipes and blood vessels. Here, we reveal the effects of flow fluctuations on the development of *Pseudomonas putida* biofilms through systematic microfluidic experiments and the development of a theoretical model. Our experimental results showed that biofilm growth under fluctuating flow conditions followed three phases: lag, exponential, and fluctuation phases. In contrast, biofilm growth under steady-flow conditions followed four phases: lag, exponential, stationary, and decline phases. Furthermore, we demonstrated that low-frequency flow fluctuations promoted biofilm growth, while high-frequency fluctuations inhibited its development. We attributed the contradictory impacts of flow fluctuations on biofilm growth to the adjustment time (*T*_0_) needed for biofilm to grow after the shear stress changed from high to low. Furthermore, we developed a theoretical model that explains the observed biofilm growth under fluctuating flow conditions. Our insights into the mechanisms underlying biofilm development under fluctuating flows can inform the design of strategies to control biofilm formation in diverse natural and engineered systems.

## Introduction

Biofilms are consortiums of bacterial cells stuck together by extracellular polymeric substances (EPSs)^1,2^. They are ubiquitous in rivers^3–6^, coastal areas^7^, drinking water distribution systems (DWDS)^8–10^, and human organs^11^. Biofilms have also been utilized for degrading polycyclic aromatic hydrocarbons (PAHs)^12^, enhancing oil recovery efficiency^13^, and removing excess nutrients and contaminants from wastewater^14^. Biofilm thickness is a key parameter controlling bacterial clogging and the efficacy of biofilm-based bioremediation projects^15,16^. For example, in moving bed biofilm reactors (MBBR) in wastewater treatment plants, biofilms with a desired thickness of around 100 μm have shown to have the optimum contaminant-removing efficiency due to efficient mass transfer^17^. Fundamental understanding and quantitative characterization of physical factors that control biofilm thickness are critical to predicting and managing biofilms, but they are currently lacking.

Hydrodynamic conditions, such as flow velocity and shear stress, are known to impact the development, or the time evolution of biofilm properties^18–22^. However, the majority of current studies have focused on steady flow, namely time-invariant constant flow, despite the fact that fluctuating flows are common in various natural and industrial environments, such as in rivers due to rainfalls and droughts^23,24^, in pipes due to intermittent water usage^25,26^, and in circulatory systems due to pulsating heartbeat^27,28^. Fluctuating flows can impact biofilm development because the oscillatory components of fluctuating flows alter the real-time distribution of pressure, velocity, and shear stress, as well as mixing and nutrient transport rates^29^. Some studies show that fluctuating flow controls the biological and chemical properties of biofilms. For example, Timoner et al. found that fluctuating flows increased the functional diversity of biofilms^30^. Kooij et al. cultivated *Legionella pneumophila* biofilms on metallic materials and reported that fluctuating flow promoted biofilm formation rate^31^. Preciado et al. found that shorter intermittent periods led the biofilm to produce a higher proportion of aromatic organic compounds^32^. Despite the recognition of the important control of flow fluctuations on the biological and chemical characteristics of biofilms, there is a lack of knowledge on how flow fluctuations regulate biofilm thickness and morphological structures.

Here, we investigated the impacts of fluctuating flow on the thickness and morphological structures of *Pseudomonas putida* biofilms through microfluidic experiments and proposed a theoretical model to account for such impacts. We chose *P. putida* as a model micro-organism because it is commonly found on the surfaces of sediment^33^, soils^34^ and drinking water systems^35^. *P. putida* has been widely used in bioremediation due to its ability to degrade a wide range of contaminants^12^, such as lignin^36^, heavy metals^37^, and phenols^38^. We grew *P. putida* biofilms in microfluidic channels under fluctuating flows of frequencies ranging from 2 × 10^−5^ to 1 × 10^−3^ Hz and a steady flow with the same mean shear stress. We imaged biofilms using a Confocal Laser Scanning Microscope (CLSM) during the course of biofilm growth. Based on these images, we calculated the thickness and areal coverage of biofilms as a function of growth time and quantified the impacts of flow fluctuations on *P. putida* biofilm growth. Furthermore, we developed a theoretical model that explains how flow fluctuations impact biofilm development. Our experimental results and theoretical model provide a foundation for future prediction and control of biofilm development via controlling the frequency of fluctuating flows.

## Results

### Biofilm development on the sidewalls and top surfaces of the microfluidic channel

**Fig. 1:**
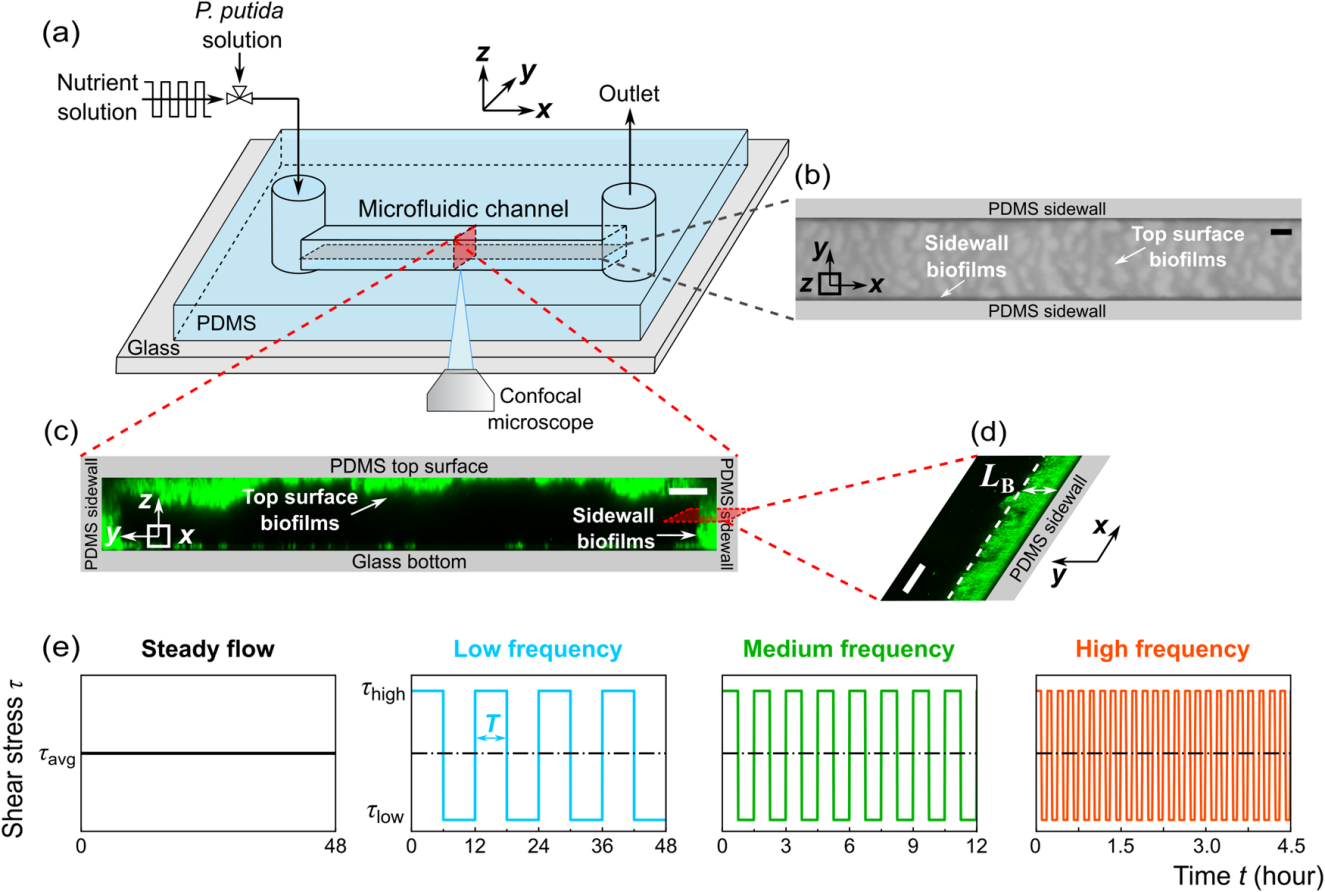
Development of *Pseudomonas putida* biofilms in a microfluidic channel under fluctuating flow conditions. **a** Schematic diagram of the experimental setup to observe the development of biofilms in a microfluidic channel. The *x*, *y*, and *z* axes denote the flow direction, lateral direction, and vertical direction, respectively. **b** Microscopic image of biofilms, using the transmitted detector (TD) function of the confocal microscope, in the horizontal cross-sectional plane of the channel, indicated by the gray color in (**a**). The scale bar is 100 μm. The biofilm-related parameters were measured from the TD images. **c** 3D images of the biofilms stained by a nucleic acid staining dye (SYTO-9), using the fluorescence detector of the confocal microscope, in the red vertical *y*-*z* plane in (**a**). The scale bar is 25 μm. **d** A zoomed-in confocal image showing biofilms on the sidewalls of the channel in the *x*–*y* plane. *L*_B_ denotes the average biofilm thickness on the sidewall of the channel. The scale bar is 25 μm. **e** The nutrient solution was injected into the microfluidic channel for 48 h under four flow conditions: steady-flow (black line, frequency *f* = 0 Hz), low-frequency fluctuating flow (cyan line, frequency *f* = 2×10^-5 ^Hz, the duration for low or high shear stress *T*_LF_ is 6 h), medium-frequency fluctuating flow (green line, *f* = 2×10^-4 ^Hz, *T*_MF_ = 45 min), and high-frequency fluctuating flow (orange line, *f* = 1 × 10^−3^ Hz, *T*_HF_ = 5.6 min). The average, low, and high shear stress is *τ*_avg_ = 3.5 Pa, *τ*_low_ = 0.05 Pa, and *τ*_high_ = 6.9 Pa, respectively.

To investigate the impacts of flow fluctuations on the growth of *Pseudomonas putida* biofilms, we grew the *P. putida* biofilms in a straight microfluidic channel under four flow conditions: a steady-flow (frequency *f* = 0 Hz) and fluctuating flows at three frequencies, i.e., low-frequency (LF, *f* = 2 × 10^−5^ Hz), medium-frequency (MF, *f* = 2 × 10^−4^ Hz), and high-frequency (HF, *f* = 1 × 10^−3^ Hz). The mean shear stress of these four flow conditions was the same for all cases, specifically *τ*_avg_ = 3.5 Pa (see “Methods” for details). For the fluctuating flows, the low shear stress and high shear stress were *τ*_low_ = 0.05 Pa and *τ*_high_ = 6.9 Pa, respectively. The low shear stress is below the critical shear stress (*τ*_crit-flat_ = 0.3 Pa) for early-stage *P. putida* biofilms to grow on flat surfaces^39^, while the high shear stress is above the critical shear stress. The rectangular microfluidic channel was made of polydimethylsiloxane (PDMS), a gas permeable material, bonded to a glass coverslip (Fig. 1a). Note that *τ*_avg_ = 3.5 Pa was the averaged shear stress over the whole channel. The average wall shear stress on the top/bottom surfaces was 4.4 Pa, higher than the average shear stress 2.6 Pa on the sidewalls (Supplementary Method 1).

During the biofilm development experiments, we first seeded the channel with *P. putida* (wild type) cells and then injected nutrient solution (M9 solution with 1% d-glucose as the carbon source) into the channel (see “Methods” for details). Over the course of biofilm development, we imaged the biofilms in the middle-depth plane of the channel periodically using the transmitted detector (TD) function of Confocal Laser Scanning Microscope (Fig. 1b and Supplementary Fig. 1). The biofilm-related parameters were calculated from these TD images (see “Methods” for details). In addition, for selected replicate experiments, we stained the biofilms using the nucleic acid dye SYTO-9 after 24-h growth time and imaged the three-dimensional distribution of the fluorescence of the biofilms using the confocal microscope (see “Methods” for details). Figure 1c shows a representative 3D structure of the stained biofilms. For the experimental conditions considered here, we observed two types of biofilms: membrane-like thin-film structures with relatively uniform thickness on the sidewalls of the channel (Fig. 1d) and heterogeneous ripple-like biofilms on the top PDMS surfaces of the channel (Fig. 1b, c). Planktonic cells were also observed outside the biofilms in the microfluidic channel under low shear stress (Supplementary Fig. 2).

Compared with noticeable biofilms on the top and side PDMS surfaces, only sparsely distributed cell aggregates were observed on the bottom glass coverslip instead of biofilms (as suggested by the 3D imaging in Fig. 1c). While the shear stress on the top and bottom surfaces is the same, the cell density on the bottom glass surface was 50% smaller than that on the top PDMS surface (Supplementary Method 2 and Supplementary Fig. 3). The fact that a lower number of cells were observed on the bottom glass surface and they did not form biofilms suggests that *P. putida* cells preferably attached to the PDMS and then formed biofilms preferentially on PDMS surfaces instead of glass surfaces. The preferential attachment of *P. putida* cells to PDMS surfaces, also observed in another study^40^, is likely because the bottom glass coverslip has negative surface charges, whereas the PDMS surface is close to neutral^41,42^, making PDMS a better surface for the negatively charged bacteria *P. putida* to attach to and further form biofilms^43^.

### Impacts of flow fluctuations on the thickness of biofilms on the sidewalls

**Fig. 2:**
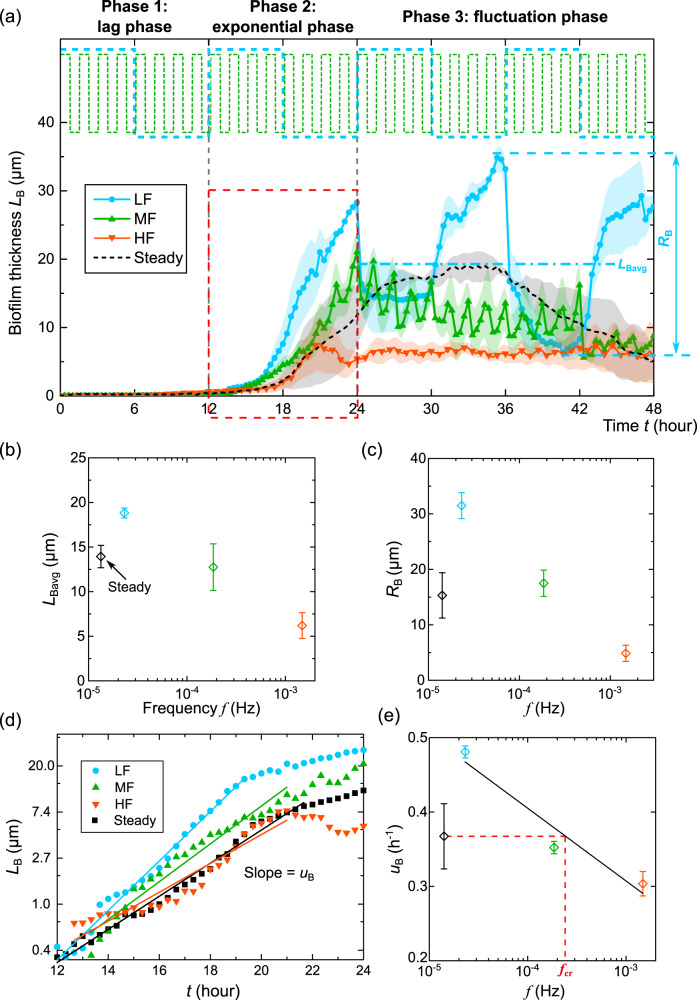
Impacts of flow fluctuations on the thickness of biofilms on the sidewall of the microfluidic channel. **a** The time evolution of biofilm thickness *L*_B_ on the sidewalls. The shadows of the lines represent the standard errors of the mean from three replicate experiments. The gray vertical dashed lines divide the three growth phases. The horizontal dot-dashed lines indicate the average thickness (*L*_Bavg_) of biofilms during the fluctuation phase. The amplitude of biofilm thickness (*R*_B_) is defined as the difference between the maximum and minimum biofilm thickness in the fluctuation phase. The red dashed box highlights the data used in (**d**). The top part of the figure shows the temporal profile of shear stress (Fig. 1e). **b** The average biofilm thickness (*L*_Bavg_) during the fluctuation phase under steady flow and fluctuating flow conditions. The black, cyan, green, and orange symbols represent the data of steady flow and low, medium, and high-frequency fluctuating flow, respectively (same for (**c**–**e**)). **c** The amplitude of biofilm thickness (*R*_B_) under steady flow and fluctuating flow conditions. **d** The biofilm thickness measurements over growth time during the exponential phase (the red dashed box in (**a**)) were linearly fitted on a semi-logarithmic scale. The slope of the fit represented the average growth rate (*u*_B_) of the biofilm thickness. **e** The growth rate of biofilm thickness (*u*_B_) for steady flow and fluctuating flow conditions. *f*_cr_ represents the critical frequency above which the flow fluctuation inhibits biofilm growth. The error bars for (**b**, **c**, and **e**) indicate the standard error of the mean from three replicate experiments.

To reveal the impacts of flow fluctuations on biofilm development, we investigated the impacts of the flow fluctuating frequency on the average thickness of the membrane-like biofilms formed on the sidewalls of the microfluidic channel (Fig. 1d), hereafter referred to as the biofilm thickness. Specifically, we compared the biofilm thickness under the steady-flow condition with that under fluctuating flow conditions with three fluctuating frequencies (Fig. 1e). The average shear stress (averaged over the whole channel) for each flow condition was the same, *τ*_avg_ = 3.5 Pa. The biofilm thickness, calculated in the *x*–*y* plane (Fig. 1d) on the sidewalls, was plotted as a function of time (*t* is the time since the flow started) in Fig. 2a. For the steady-flow condition with a constant shear stress *τ*_avg_ = 3.5 Pa, we observed that the time evolution of biofilm thickness followed four phases: (1) a lag phase (before 12 h), when no visible biofilms were observed; (2) an exponential phase (12–24 h), when the biofilm thickness increased exponentially with time; (3) a stationary phase (24–36 h), when the biofilm thickness reached a plateau; and (4) a decline phase (after 36 h). The observed four phases are consistent with the four phases of the biofilm life cycle described in previous studies^44,45^.

Compared with the four phases of biofilm development under a steady-flow condition, the thickness of sidewall biofilms in fluctuating flows followed three phases: (1) a lag phase (before 12 h) similar to the steady flow condition; (2) an exponential phase (12–24 h) similar to the steady flow condition, when the biofilm thickness increased exponentially with time for all the frequencies considered here, and no obvious biofilm detachment was observed; and (3) a fluctuation phase (after 24 h), when the biofilm thickness fluctuated around a mean value, indicating a dynamic equilibrium of biofilm growth and detachment^46^. During the fluctuation phase, we observed that pieces of the biofilms on the sidewalls detached locally immediately after the shear stress switched from low to high (Supplementary Fig. 4 and Supplementary Video 1). When the shear stress switches from high to low, biofilms will regrow in these detached regions (Supplementary Fig. 4). These localized detachment and regrowth lead to a net equilibrium in the biofilm thickness. In addition, we determined the dominant frequency of the biofilm thickness versus time signal using Fast Fourier Transform (FFT) analysis. We found that the dominant frequency of biofilm thickness variations in the fluctuation phase is similar to the frequency of the fluctuating flow (Supplementary Fig. 5), confirming that the changes in biofilm thickness during the fluctuation phase are due to the variation of the shear stress with time.

To evaluate the impact of pressure change due to flow fluctuations on the biofilm thickness, we calculated the flow-induced pressure in the channel using Hagen-Poiseuille equation^47^: $p = \frac{{12\,Ql\mu }}{{wh^3}}$, where *Q* is the flow rate, *l* is the channel length, *μ* is the dynamic viscosity of water, *w* is the channel width, and *h* is the channel depth. The change in pressure when the flow switched from high to low was $\Delta P = \frac{{12\Delta Ql\mu }}{{wh^3}} \approx 4\,{{{\mathrm{Pa}}}}$, over four orders of magnitude smaller than the atmospheric pressure (101 kPa) that the biofilms experienced. Therefore, we conclude that the change in biofilm thickness when the flow fluctuates is not due to the flow-induced pressure. Instead, our observation of local detachment of biofilms when the shear stress switched from low to high (Supplementary Fig. 4) suggests that the decrease in biofilm thickness is due to shear-induced biofilm detachment.

To rule out the possibility that flow fluctuations impact biofilm development by altering oxygen and nutrients concentration, we calculated the diffusion and replenishment timescales of oxygen and nutrients into the microchannel (Supplementary Method 3). The diffusion timescales for oxygen and nutrients were estimated to be 0.9 and 3.2 s, respectively. The longest replenishment time, occurring during low flow conditions, for the new nutrient to fully replace the channel was calculated to be 7.2 s. These timescales are much smaller than the shortest time interval of the fluctuating flows, 5.6 min, suggesting that oxygen and nutrients remain abundant during the experiments and are not the limiting factors of biofilm growth. Furthermore, we conducted biofilm growth experiments at two additional glucose concentrations, half and two times the current concentration, and observed that the biofilm growth followed a similar trend (Supplementary Fig. 6). This suggests that the nutrient supply in our experiments is abundant.

To further quantify the impacts of flow fluctuations on the thickness of sidewall biofilms, we calculated the average biofilm thickness (*L*_Bavg_) during the fluctuation phase and the amplitude of biofilm thickness (*R*_B_), defined as the difference between the maximum and minimum biofilm thickness during the fluctuation phase (see Fig. 2a–c). For comparison, we calculated *L*_Bavg_ and *R*_B_ for steady flow during 24–48 h (which corresponds to the fluctuation phase), which are *L*_Bavg_ = 14 μm and *R*_B_ = 15 μm, respectively. For the flow with low-frequency fluctuation (*f* = 2 × 10^−5^ Hz), *L*_Bavg_ and *R*_B_ are 19 μm and 31 μm, which are 35% and 106% higher than those for the steady-flow, respectively. Compared with the increase in *L*_Bavg_ and *R*_B_ with increasing frequency in the low-frequency range (*f* = 0 to 2 × 10^−5^ Hz), *L*_Bavg_ and *R*_B_ decreased with frequency from *f* = 2 × 10^−5^ to 2 × 10^−4^ Hz. Specifically, as frequency increased to 2 × 10^−^^4 ^Hz, *L*_Bavg_ = 13 μm and was 9% lower than that for the steady-flow. As the frequency further increased to *f* = 1 × 10^−3 ^Hz, *L*_Bavg_ and *R*_B_ decreased to 6 μm and 5 μm, respectively, corresponding to 56% and 68% lower than that for the steady flow, respectively. Overall, these observations suggest that low flow fluctuations (e.g., *f* < 2 × 10^−5^) promote biofilm growth, while high-frequency fluctuations (e.g., *f* > 1 × 10^−3^) inhibit biofilm growth.

To further confirm the contradictory roles of flow fluctuations on biofilm growth, we fitted the biofilm thickness versus growth time during the exponential phase linearly using the least squares method, as shown in Fig. 2d. We calculated the slope of the fit as the effective growth rate (*u*_B_) of the biofilm thickness. As shown in Fig. 2e, in the low-frequency range, as flow frequency increased from 0 (the steady-flow condition) to 2 × 10^−5^ Hz, *u*_B_ increased by 31%. In the high-frequency range, *u*_B_ decreased by 4% and 17% under medium- and high-frequency conditions (*f* = 2 × 10^−4^ and 1 × 10^−3^ Hz) compared with steady-flow condition. These observations suggest that low flow fluctuations (*f* < 2 × 10^−5^ Hz) increase biofilm growth rate during the exponential phase, while high flow fluctuations (*f* > 1 × 10^−3^ Hz) inhibit biofilm growth.

Furthermore, from the linear fit, we determined the critical frequency *f*_cr_ = 2.4 × 10^−4^ Hz, for which the biofilm growth rate is equal to the steady-flow condition (Fig. 2e). This critical frequency represents the threshold below which biofilm growth is promoted and above which biofilm growth is inhibited. This critical value can potentially be applied to control biofilm growth for various applications, including bioreactor optimization, wastewater treatment, and medical device design.

### A theoretical model to predict biofilm growth under fluctuating flow conditions

**Fig. 3:**
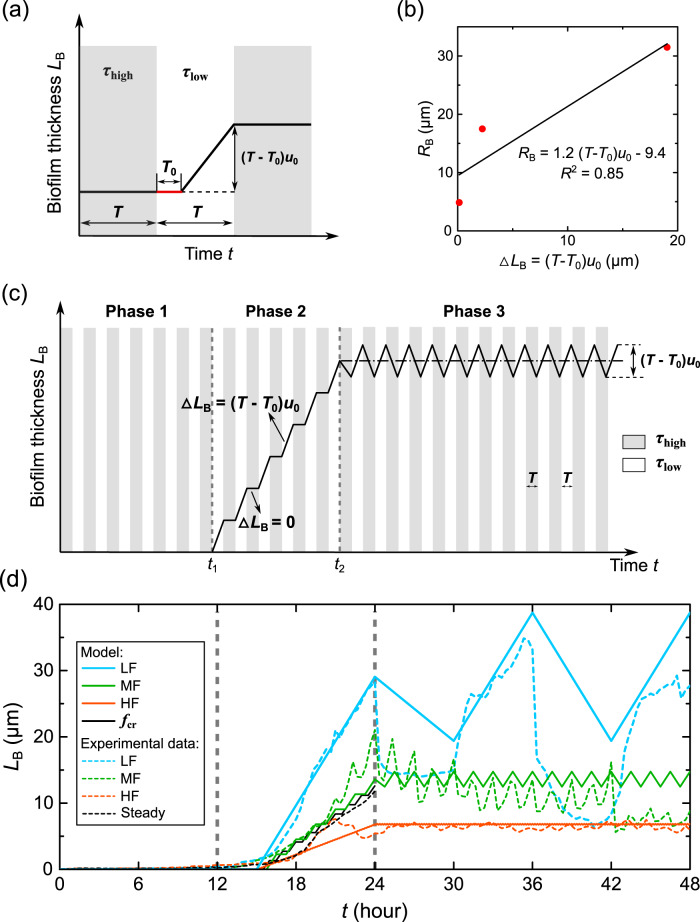
A theoretical model to predict biofilm growth and its comparison with experimental measurements. **a** The biofilm growth during the exponential phase. The gray shadowed areas show the intervals of high shear stress (*τ*_high_ = 6.9 Pa), and the white areas show the intervals of low shear stress (*τ*_low_ = 0.05 Pa). The duration of each low and high shear stress interval is *T*, as shown in Fig. 1e. *u*_0_ is the growth rate of the biofilm thickness at low shear stress. *T*_0_ is the adjustment time for bacteria to adjust to the flow and form biofilms. **b** The amplitude of the measured biofilm thickness *R*_B_ in the fluctuating phase versus the net growth of biofilm thickness ∆*L*_B_ = (*T* − *T*_0_)*u*_0_. **c** The proposed biofilm growth model (Eqs. (1) to (3)). The flow fluctuates with a medium frequency *f* = 2 × 10^-4 ^Hz, as indicated by the green lines in Fig. 1e. The gray vertical dashed lines divide the three growth phases: lag, exponential, and fluctuation phases. **d** Comparison of the proposed growth model (solid lines) with measured biofilm thickness (dashed lines, as shown in Fig. 2a). *f*_cr_ represents the critical frequency above which the flow fluctuation inhibits biofilm growth.

To explain the observed impacts of flow fluctuations on biofilm development, we developed a theoretical model to predict biofilm growth based on the following hypotheses: (1) low-frequency fluctuations promote biofilm growth as biofilms grow faster during the low shear stress periods of the flow, and (2) high-frequency fluctuations inhibit biofilm growth because biofilms need time (the adjustment time *T*_0_) to adapt to the flow transition (Fig. 3a). The first hypothesis reflects the observation of our previous study^39^ that low shear stress increases early-stage biofilm growth, while high shear stress inhibits its growth. Here, the high shear stress in our study (*τ*_high_ = 6.9 Pa) exceeds the critical shear stress for early-stage *P. putida* biofilms to grow (*τ*_crit-flat_ = 0.3 Pa), so we assume biofilms only grow during the low shear stress (*τ*_low_ = 0.05 Pa) periods during the exponential phase^39^. The second hypothesis reflects the observation that biofilms need some time to adjust to the flow and grow^48,49^.

Building on these hypotheses, we developed a theoretical model for biofilm growth under fluctuating flows, assuming that biofilms only grow during the low shear stress period with an adjustment time (*T*_0_) in the exponential growth phase. Specifically, we divided the biofilm growth period into three phases: lag, exponential, and fluctuation phases. For the lag phase (*t* = 0–12 h), we assume no biofilm growth because no obvious biofilms were observed in experiments, namely:

1 $$L_{{{{\mathrm{B}}}} - {{{\mathrm{phase}}}}1} = 0$$

During the exponential phase (*t* = 12–24 h), we observed intermittent increases in biofilm thickness, i.e., biofilm thickness increased under low shear stress but remained relatively constant or decreased slightly during high shear stress (Supplementary Fig. 7). To describe this growth behavior during the exponential phase, we proposed a simplified growth model assuming that biofilms do not grow during intervals of high shear (*τ*_high_ = 6.9 Pa) and grow at a constant rate *u*_0_ after adjustment time *T*_0_ during low shear stress intervals (*τ*_low_ = 0.05 Pa) (Fig. 3a). The adjustment time *T*_0_ represents the time for bacteria to adapt to the low flow condition and grow biofilms after the shear stress changed from high to low^48,49^. The biofilm growth rate during the low shear stress intervals, *u*_0_ = 3.2 μm/h, was determined by fitting the biofilm thickness against growth time linearly during the low shear stress interval (18–24 h) for the low-frequency case (Supplementary Fig. 8). We assume that biofilms grow linearly with growth rate *u*_0_ after *T*_0_, so the net growth in biofilm thickness after one low-high shear stress interval is (*T* − *T*_0_)*u*_0_. *T* is the duration of each low or high shear stress interval. If there are *n* low-high shear stress cycles during the exponential phase, then the total biofilm thickness after the exponential phase is

2 $$L_{{{{\mathrm{B}}}} - {{{\mathrm{phase}}}}2} = n\Delta L_B$$

where *∆L*_B_ = (*T* − *T*_0_)*u*_0_ is the growth of biofilm thickness after one low shear stress (*τ* = *τ*_low_) interval, and ∆*L*_B_ = 0 under high shear stress (*τ* = *τ*_high_) intervals. *n* is the number of low-high shear stress cycles during the exponential growth period. For the high-frequency flow (*f* = 1 × 10^−3^ Hz, *T*_HF_ = 5.6 min), the number of cycles during the exponential growth phase is *n* = 48, and the biofilm thickness after the *n* growth cycles is *L*_Bavg-HF_ = 6.4 μm. By substituting *L*_Bavg-HF_, *n*, and *T*_HF_ into Eq. (2): *L*_Bavg-HF_ = *n*(*T*_HF_ − *T*_0_)*u*_0_, we found *T*_0_ = 3 min (Supplementary Method 4). The 3-min adjustment time for biofilm to start growing is consistent with a previous study that showed the biofilm formed on surfaces exposed to seawater within the first few minutes^50^.

During the fluctuation phase (*t* = 24–48 h), we observed that the biofilm thickness fluctuated up and down around a steady mean value. Specifically, we observed an increase in biofilm thickness during low shear stress and a decrease by the same amount during high shear stress, indicating a dynamic equilibrium between growth and detachment within the biofilm. The decrease in biofilm thickness during high flow in the fluctuating phase is likely contributed by the local detachment of biofilms when the shear stress switched from low to high, while their increase during low flow corresponds to biofilm regrowth (Supplementary Fig. 4). In our model, we simplified this cyclic behavior as a fluctuation around a mean value equal to the thickness at the end of the second phase (*L*_B-phase2_). We found that the amplitude of the biofilm thickness fluctuation (*R*_B_) is correlated linearly with the net growth of biofilm thickness during one low shear stress interval (Fig. 3b), i.e., ∆*L*_B_ = (*T* − *T*_0_)*u*_0_. Therefore, we assumed that the amplitude of the biofilm thickness fluctuation is equal to (*T* − *T*_0_)*u*_0_ in our model. Thus, the biofilm thickness during the fluctuation phase can be written mathematically as:

3 $$L_{{{{\mathrm{B}}}} - {{{\mathrm{phase}}}}3}\left( t \right) = L_{{{{\mathrm{B}}}} - {{{\mathrm{phase}}}}2} + \frac{{\left( {T - T_0} \right)u_0}}{\pi }{{{\mathrm{arcsin}}}}\left( {{{{\mathrm{sin}}}}\left( {\frac{\pi }{{{{\mathrm{T}}}}}\left( {t - t_2 - \frac{T}{2}} \right)} \right)} \right)$$

where *t*_2_ = 24 h is the start time of the fluctuation phase.

To evaluate the validity of our proposed biofilm growth model (Eqs. (1) to (3), Fig. 3c), we compared the biofilm thickness predicted by our model with experimental measurements shown in Fig. 2a. As shown in Fig. 3d, the model predictions are consistent with the biofilm thickness at the lag phase, exponential phase, and fluctuation phase of the biofilm growth for all the fluctuating frequencies considered here (2 × 10^−5^ < *f* < 1 × 10^−3 ^Hz). The root-mean-square error (RMSE) values for low-frequency, medium-frequency, and high-frequency experiments were 7.0 μm, 3.5 μm, and 0.9 μm, respectively. The coefficient of determination (*R*^2^) for low-frequency, medium-frequency, and high-frequency cases were 0.63, 0.60, and 0.72, respectively. The relatively low RMSE values (7.0 μm, 3.5 μm, and 0.9 μm) and relatively high *R*^2^ values (0.63, 0.60, and 0.72) suggest that our model is able to capture the influence of flow fluctuations on biofilm growth. The agreement also confirms our hypothesis that the inhibition of biofilm growth under high-frequency flow fluctuation is due to the adjustment time (*T*_0_) needed for biofilms to start growing after the shear stress switched from high to low.

Moreover, we predicted the biofilm growth at the critical frequency (*f*_cr_ = 2.4 × 10^−4^ Hz) when the biofilm growth rate was equal to the growth rate in the steady flow, as indicated in Fig. 2e. We compared the predicted biofilm thickness at this critical frequency with the steady-flow experimental measurements and found a good agreement between them (black dashed and solid lines in Fig. 3d). This agreement further indicates that our developed model can predict the biofilm growth behavior under different fluctuating flow frequencies and facilitate future utilization of the flow frequency to control the biofilm growth.

### Impacts of flow fluctuations on biofilm growth on the top surface

**Fig. 4:**
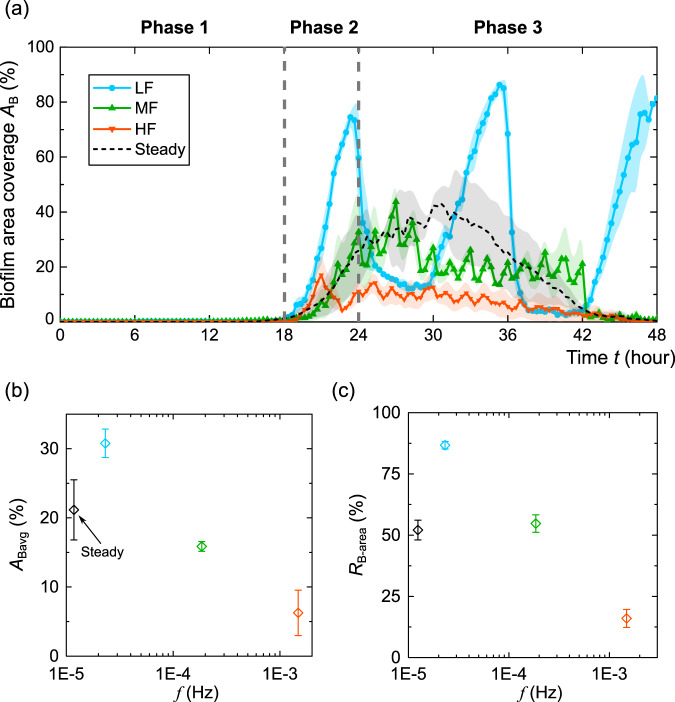
Impacts of flow fluctuations on the areal coverage of biofilms on the top surface of the microfluidic channel. **a** The biofilm coverage on the top PDMS surface of the microfluidic channel as a function of time for steady flow and fluctuating flow conditions indicated in Fig. 1e. The circular, triangular, and square dots indicate the average values of biofilm coverage of three replicate experiments. The shadows of the lines represent the standard errors from three replicated experiments. The gray vertical dashed lines divide the three growth phases: lag, exponential, and fluctuation phases. **b** The average areal coverage of biofilms (*A*_Bavg_) during the fluctuation phase under steady flow and fluctuating flow conditions. The black, cyan, green, and orange symbols represent the data of steady flow and low-, medium-, and high-frequency fluctuating flow, respectively (same for (**c**)). **c** The amplitude of the areal coverage of biofilms (*R*_B-area_) under steady flow and fluctuating flow conditions. The error bars for (**b**, **c**) indicate the standard error of the mean from three replicate experiments.

In the above sections, we revealed the impacts of flow fluctuations on the growth of membrane-like biofilms on the sidewalls of the microfluidic channel. In addition to the sidewalls, biofilms were also observed on the top PDMS surfaces of the microfluidic channel (Fig. 1c). Compared with the relative uniform distribution of the sidewall biofilms, the biofilms on the top surfaces exhibit heterogeneously-distributed ripple-like structures (Fig. 1b). To demonstrate how flow fluctuations impact the development of biofilms on the top surface, we calculated the areal coverage of biofilms (*A*_Bavg_) on the top surfaces as a function of time under the four flow conditions considered above, namely steady flow and three fluctuating flows with low, medium, and high frequencies. As shown in Fig. 4a, the temporal evolution of the biofilm areal coverage on the top surfaces followed similar phases as the sidewall biofilms (Fig. 2a), specifically lag, exponential, and fluctuation phases. The lag phase of the ripple-like biofilms on the top surfaces is about 18 h, longer than the 12 h lag phase of biofilm development on the sidewalls. This is likely because the cross-section of the channel is a narrow rectangular, so the shear stress is higher on the top surfaces (4.4 Pa) than on the sidewalls (2.6 Pa). A high shear stress hinders biofilm growth^39^ and thus may delay the start of the exponential phase. In addition, we observed a steep decrease in biofilm coverage on the top surface after 42 h in medium- and high-frequency conditions (Fig. 4a). Such decrease is similar to the decrease in biofilm thickness after 36 h for the steady-flow condition (black line in Fig. 2a), which is often referred to as the decline phase of the biofilm life cycle^44^. In contrast, at the low-frequency fluctuation condition (*f* = 2 × 10^−5 ^Hz, *T*_LF_ = 6 h), we didn’t observe a steep decrease during our 48 h experimental duration, suggesting that low-frequency flow fluctuations can elongate the biofilm life cycle. The duration of the low-shear-stress interval for the low fluctuation case is *T*_LF_ = 6 h, comparable to the exponential growth period of *P. putida* biofilms (8–12 h)^39^. In contrast, the durations of low-shear-stress intervals for the medium- and high-frequency cases are *T*_MF_ = 45 min and *T*_HF_ = 5 min, respectively, much smaller than the biofilm exponential growth period. We hypothesize that low-frequency fluctuations, with duration of low-shear-stress interval comparable to biofilm growth time, can elongate the biofilm life cycle. This is likely because such duration would allow biofilms in the nutrient-depleted region to regrow to the exponential phase, though more studies need to be done to confirm this hypothesis.

In addition to instantaneous biofilm areal coverage, we calculated the average biofilm areal average (*A*_Bavg_) during the fluctuation phase (*t* = 24–48 h) and the magnitude of the fluctuation *R*_B-area_, which is equal to the difference between the maximum and minimum biofilm thickness during the fluctuation phase. The resultant values as a function of flow fluctuation frequencies are shown in Fig. 4b, c. As shown in these figures, the flow fluctuations have similar impacts on the biofilm growth on the top surfaces as on the sidewalls. Under steady-flow conditions, the average biofilm areal coverage *A*_Bavg_ during the fluctuation phase was 0.21, with an amplitude *R*_B-area_ of 0.52. Under low-frequency fluctuation (*f* = 2 × 10^−5^ Hz), compared to the steady-flow case, *A*_Bavg_ increased by 45% to 0.30, with *R*_B-area_ increasing by 66% to 0.86. Under medium-frequency fluctuation (*f* = 2 × 10^−4^ Hz), *A*_Bavg_ decreased by 25% to 0.16, and *R*_B_ decreased by 5% to 0.54, compared to steady-flow conditions. The high-frequency fluctuation (*f* = 1 × 10^−3^ Hz) led to a substantial decrease of 70% in *A*_Bavg_ to 0.06, with *R*_B_ decreasing by 69% to 0.16, compared to steady-flow conditions. The above results show that, similar to the biofilms on sidewalls, the biofilm growth on the top surfaces is also promoted at low frequency (*f* < 2 × 10^−5 ^Hz) and inhibited at high frequency (*f* > 1 × 10^−3 ^Hz). To further reveal the impacts of flow fluctuations on the pattern of ripple-like biofilms, we calculated the ripple wavelength^51^ (defined as the distance between the centers of two adjacent ripple-like biofilms) and the ripple number (defined as the number of ripples in the field of view) (Supplementary Fig. 9). Our results, as shown in Supplementary Fig. 9, demonstrate that flow fluctuations exhibit similar effects on the ripple wavelength, with larger values observed under low-frequency conditions and smaller values under high-frequency conditions. In contrast, the ripple number did not show a clear trend with increasing flow frequencies.

## Discussion

In this study, we quantified the impacts of flow fluctuations on biofilm development through direct visualization of biofilm growth in microfluidic devices. Our results show that under fluctuating flow conditions, biofilm growth followed three phases: lag, exponential, and fluctuation phases, which are different from the four phases of biofilm growth under steady flows: lag, exponential, stationary, and decline phases. Moreover, we show that under low-frequency fluctuation conditions (*f* < 2 × 10^−5 ^Hz), the biofilm growth rate and the mean biofilm thickness at the fluctuation phase are larger than in the steady-state condition (*f* = 0 Hz). In contrast, under high-frequency fluctuation conditions (*f* > 1 × 10^−3 ^Hz), the biofilm thickness decreased with increasing frequency. Our theory suggests that the inhibition of biofilm growth by high-frequency fluctuations is because biofilms need an adjustment time (*T*_0_) to grow after shear stress switches from high to low, and thus the time for biofilm to grow during low shear stress is limited.

Furthermore, we developed a theoretical model that predicted the growth of biofilm thickness under fluctuating flow conditions with varying frequencies. The proposed model successfully predicted the biofilm thickness at the lag phase, exponential phase, and fluctuation phase of biofilm growth. For the prediction of the exponential phase, our model attributes the fluctuation effects to the net growth of biofilm thickness during each low shear stress interval, which is ∆*L*_B_ = (*T* − *T*_0_)*u*_0_. In this equation, *T*_0_ = 3 min represents the adjustment time required for the biofilms to adjust to the flow transition. *u*_0_ denotes the growth rate during the low shear stress condition. Note that in this study, the high shear stress (*τ*_high_ = 6.9 Pa) is much larger than the critical shear stress (*τ*_crit-flat_ = 0.3 Pa) for early-stage *P. putida* biofilms to grow^39^, thus, we assumed biofilms do not grow during the high shear stress condition. Therefore, our model may not be suitable for conditions when the high shear stress is below the critical shear stress and biofilms continue to grow under high shear stress^52^. In the fluctuation phase, we observed that biofilms experienced a dynamic equilibrium of detachment and regrowth. The detachment of biofilms during high flow fluctuations was attributed to local detachment, and their increase by the same amount during low flow was attributed to biofilm regrowth, as shown in Supplementary Fig. 4. The local detachment and regrowth of biofilms lead to a net equilibrium in the biofilm thickness. An adjustment time *T*_0_ is needed for biofilm to regrow after the flow switch, likely because the detachment in the outer layer of the biofilm that is actively growing exposes an underlying nutrient-depleted layer of the biofilms^53^, which may require an adjustment time *T*_0_ to regrow. Our model simplified this cycle by assuming that the biofilm thickness fluctuates around a mean value with an amplitude of ∆*L*_B_ = (*T* − *T*_0_)*u*_0_. Our model predicted biofilm growth at a wide range of frequencies (2 × 10^−5^ < *f* < 1 × 10^−3 ^Hz) and is thus an efficient tool for predicting the impact of flow fluctuations on biofilm growth in diverse environments.

The shear stress (*τ*_avg_ = 3.5 Pa) here resembles the range in blood vessels (on the order of 1 Pa)^54^ and biofilm reactors (1.0–2.5 Pa)^55^. Additionally, the fluctuation periods used in this study (5.6 min to 6 h) resemble the durations of rainfall events (15 min to a few hours)^56^, the intermittent time period of the water supply system (~ 6 h)^32^, and the hydraulic retention time in the operation process of moving bed biofilm reactors (MBBR) (~10 h)^57^. Therefore, our results have potential implications for predicting and controlling biofilm development in natural and engineered systems, such as drinking water distribution systems and medical devices, where biofilms are consistently exposed to fluctuating flows^23,26,28^. The critical frequency (*f*_cr_ = 2.4 × 10^−4 ^Hz) identified in our study can be applied to control biofilm growth in these environments. Moreover, the modified biofilm growth model we developed can be used to further optimize flow conditions to control biofilm growth. Note that here we only considered a single bacterial species (*Pseudomonas putida*) and one average shear stress value (*τ*_avg_ = 3.5 Pa). To further apply our model to real-world situations, we need to consider the bacterial species, shear stress values, and substrate surfaces.

In conclusion, our study demonstrates that flow fluctuations can affect biofilm development in contradictory ways. Specifically, low flow fluctuations can promote biofilm growth, while high flow fluctuations can prevent biofilm growth. The present study provides valuable insights into the mechanisms underlying biofilm development under fluctuating flows and can inform the design of strategies to control biofilm formation in various applications.

## Methods

### Bacteria culture

The strain we used here is *Pseudomonas putida* KT-2442 (a gift from Mohamed Donia’s lab, Princeton University). The bacterial solution used to seed the microfluidic chamber was prepared following the steps described below. First, *P. putida* cells were cultured from a frozen stock by incubating them in Luria Broth (LB) solution overnight (~16 h). The culture was grown to the exponential phase in an incubator at 30 °C with 200 rpm shaking. Second, we transferred the exponential phase cells to a modified M9 solution with a fully characterized chemical composition^58^ (supplemented with 0.03 M (NH_4_)_6_(Mo_7_)_24_, 4 M H_3_BO_3_, 0.3 M CoCl_2_, 0.1 M CuSO_4_, 0.8 M MnCl_2_, 0.1 M ZnSO_4_, and 0.1 M FeSO_4_). Specifically, we centrifuged 5 mL of bacterial cultures in a 50-mL tube using a temperature-controlled incubator (ES-60, Hanchen) at 3000×*g* for 10 min, after which we removed the supernatant. The bacterial deposit was then diluted with M9 medium solution to an OD_600_ of ~0.5. d-glucose at 1 wt. % concentration was added to the M9 medium as a carbon source.

### Experimental platform

Microfluidic experiments were conducted to characterize biofilm development under fluctuating flows. Schematic diagram of the microfluidic platform is shown in Fig. 1a. The system consists of a microfluidic chip, a Confocal Laser Scanning Microscope (Nikon C2 plus), and a programmable syringe pump (PHD Ultra, Harvard Apparatus). Soft lithography was used to fabricate polydimethylsiloxane (PDMS) microfluidic chips with the assistance of the University of Minnesota Nano Center. The straight channels utilized in this study have a height of 60 μm and a width of 400 μm. The channel measures ~5 mm in length from inlet to outlet. During the experiment, the chips were placed on the stage top incubator (UNO-T-H, Okolab) at a controlled temperature (30 °C). A programmable syringe pump (PHD Ultra, Harvard Apparatus) is utilized to generate fluctuating flows of the glucose solution. According to the manual book of the Harvard pump, the pump’s longest switch time during flow transitions is reported to be 27.5 s, which is considerably shorter than the minimum fluctuation period of 5.6 min used in our study. Confocal microscopy was used to image the biofilms in the microfluidic channel with 0.31 μm/pixel resolution.

### Biofilm development experiments

Biofilm development experiments were conducted following the steps described below (Fig. 1a). First, 2 mL of nutrient solution (abiotic M9 solution containing 1 wt.% d-glucose) was pumped into the microfluidics to displace the air in the channel. Then, we switched the valve (Fig. 1a) to inject 200 μL of carbon-free M9 solutions with *Pseudomonas putida* at OD_600_ = 0.5 ± 0.03 into the microfluidic channel. The cells were incubated for 30 min to allow the initial attachment of the cells to the surfaces. The initial cell concentrations on the bottom glass surface, top PDMS surface, and sidewalls were (1.3 ± 0.2) × 10^4^ cells/mm^2^, (7.7 ± 0.8) × 10^3^ cells/mm^2^, and (2.8 ± 0.6) × 10^3^ cells/mm^2^, respectively. Afterward, we switched the valve and injected the nutrient solution into the channel at varying flow conditions using a programmable syringe pump (PHD Ultra, Harvard Apparatus). The average flow rate (*Q*_avg_) was kept constant at 63 μL/min for all the experiments, and the average shear stress over the whole channel was 3.5 Pa ($\tau _{{{{\mathrm{avg}}}}} = \frac{{{{{\mathrm{6}}}}{\it{\eta }}Q_{{{{\mathrm{avg}}}}}}}{{{\it{h}}^{{{\mathrm{2}}}}{\it{w}}}}$, where *η* is fluid dynamic viscosity, *h* = 60 μm is channel depth, and *w* = 400 μm is channel width), which is on the same magnitude as the mean wall shear stress of flowing blood^54^, shear stress in drinking water pipes^59^, and moving bed biofilm reactors (MBBR)^55^. Fluctuating flows with different flow frequencies were considered. For each flow, the average low shear stress (flow rate *Q*_low_ = 1 μL/min) and high shear stress (*Q*_high_ = 125 μL/min) over the whole channel are ${{{\mathrm{\tau }}}}_{{{{\mathrm{low}}}}}{{{\mathrm{ = }}}}\frac{{{{{\mathrm{6}}}}{\it{\eta }}Q_{{{{\mathrm{low}}}}}}}{{{\it{h}}^{{{\mathrm{2}}}}{\it{w}}}}{{{\mathrm{ = }}}}0.05$ Pa, and *τ*_high_ = 6.9 Pa, respectively (Fig. 1e). The average wall shear stress on the top/bottom surfaces was 4.4 Pa, while on the sidewalls it was 2.6 Pa, which were calculated using computational fluid dynamics (CFD) simulations (detailed information can be found in Supplementary Method 1). The frequency *f* = 1/2 *T*, *T* represents the time duration under low or high shear stress. Three frequency cases were considered in this study and denoted as low-frequency (LF, frequency *f* = 2 × 10^−5 ^Hz, the duration for low or high shear stress *T*_LF_ = 6 h), medium-frequency (MF, *f* = 2×10^-4 ^Hz, *T*_MF_ = 45 min), and high-frequency (HF, *f* = 1 × 10^−3 ^Hz, *T*_HF_ = 5.6 min), which vary by approximately three orders of magnitude, which resembles the durations of rainfall events^56^, intermittent water supply time (IWS) period^32^, and hydraulic retention time in the moving bed biofilm reactors (MBBR) operation process^57^. The average flow velocity used in our study was 43 mm/s, about three orders of magnitude larger than the swimming speed of *P. putida* cells^60^ (44 μm/s), indicating that the cells were primarily influenced by fluid shear rather than their own motility. As the biofilms grew, we captured images of the biofilms using a Confocal Laser Scanning Microscope (CLSM) at 10- to 30-min intervals, depending on the frequency. The duration of the experiment is 48 h. Each experiment was biologically repeated three times.

### Confocal microscopy visualization

During the biofilm development experiments, the microfluidic channels were visualized using a Nikon C2+ Confocal Laser Scanning Microscope (CLSM) with 0.3 μm-horizontal resolution and 0.5 μm-vertical resolution. The microfluidic channel measures 5 mm in length from inlet to outlet. We imaged the middle portion of the microfluidic channel, which corresponds to a 4 mm-long region located 0.5 mm away from the inlet and outlet (Supplementary Fig. 10). During the biofilm development experiments, we employed the transmitted detector (TD) function of a Confocal Laser Scanning Microscope (Fig. 1b and Supplementary Fig. 1) to periodically capture images of the biofilms at the middle-depth plane of the channel. The biofilm-related parameters were calculated from the TD images. The wavelength of the laser used here is 488 nm. Biofilms in the entire microfluidic channel were scanned piece by piece, with each image measuring 2048 by 2048 pixels. These images were combined into a single image using the Large-Image function of the Nikon NIS-Elements software. Cross-sectional images of biofilms at the middle depth of the channel were used in our analysis. The objective magnification was 10-X and 20-X. The *P. putida* strains used here are wild type without fluorescent genes. During the experiments, the images were scanned at 10- to 30-min intervals over 48 h and saved on an HP-Z4-G4 workstation. The camera used here is a CoolSnap-DYNO (Photometrics, USA), and the exposure time is 75 ms.

To visualize the three-dimensional (3D) distribution of biofilms, we stained the nucleic acids of the biofilms for four selected replicate experiments (steady flow and fluctuating flow with low, medium, and high frequencies). Specifically, we mixed 10 μL of SYTO-9 green fluorescent nucleic acid stain (5 μM, Thermo Fisher Scientific, USA) with 10 mL of carbon-free M9 solution and then pumped the mixture into the microfluidic channel at 0.5 μL/min for 1 h during the four selected experiments^61^ (detailed procedures can be found in Supplementary Method 5). Biofilms over the channel depth were scanned at several vertical positions at 0.5 μm vertical resolution using the Z-Stack function of the Nikon NIS-Elements software. No significant decrease of the SYTO-9 signal with scanning time was observed during the imaging process. The laser used during staining is a 488 nm laser. Note that the biofilm-related parameters used in this study are derived from the 2D scan images taken at the mid-depth of the channel. This is because 3D scan of biofilms require more than 2 h (4 mm by 400 μm field of view, 60 μm depth, 0.3 μm-horizontal and 0.5 μm-vertical scanning resolution) and, as a result, cannot capture the dynamic changes in biofilms over short time intervals (5 min for our study). Our 2D scans taken at the mid-depth of the channel provide accurate measurements of the biofilm thickness because our 3D scan showed that the variation in biofilm thickness along the sidewall, across the depth of the scan, was within 3% (Supplementary Fig. 11).

### Image analysis

We scanned the biofilms in the microfluidic channel in three-dimensional space using the Z-stack function of the Nikon confocal microscope (Fig. 1). These confocal images show that the ripple-like biofilms only occupied the top surface of the channel, while biofilms on the sidewalls occupied the whole channel depth (Fig. 1b, c). Consequently, the light intensity of the pixel occupied by sidewall biofilms was darker than that of regions occupied by ripple-like biofilms on the top surface. To quantify the biofilm thickness of the sidewall biofilms and the areal coverage of ripple-like biofilms, we employed Image-J and MATLAB and followed the subsequent steps. Since the sidewall biofilms and top surface biofilms had distinct color differences (Supplementary Fig. 12a) (the pixel intensity distribution had a two-peak distribution, Supplementary Fig. 12b), we converted the grayscale images into binary images using Otsu’s method^62^ and applied this threshold to subtract the biofilm images from the background image (the image at the beginning of the experiments) (Supplementary Fig. 12c). After identifying the regions occupied by biofilms on the sidewalls, we computed the average biofilm thickness by dividing the total biofilm area by the length of the field of view (4 mm). Regarding the ripple-like biofilms formed on the top PDMS surfaces, since the ripple-like structures only occupied a portion of the space of the whole channel and had a slight color difference with the background (Supplementary Fig. 12d); the pixel intensity distribution had only one peak (Supplementary Fig. 12e) and was not suitable for using Otsu’s method^63^. Therefore, we manually determined the threshold by comparing the pixel intensity between the ripples and the background using Image-J and employed this threshold to binarize all the images (Supplementary Fig. 12f). Subsequently, we calculated the biofilm areal coverage *A*_B_ by dividing the total biofilm area by the entire area.

### Statistical analysis

The results are shown as the mean ± standard error (SE). The mean value was calculated from three replicates. The error bars indicate the standard error of three replicates. Three biological replicates were conducted for all the cases.

### Reporting summary

Further information on research design is available in the Nature Research Reporting Summary linked to this article.

### Supplementary information

Supplementary information

Reporting Summary

Supplementary Video 1

## Data Availability

All data and raw microscopic images are available in the Data Repository for University of Minnesota repository (DRUM): https://hdl.handle.net/11299/253957. The MATLAB codes for image analysis are available in the Data Repository for University of Minnesota repository (DRUM): https://hdl.handle.net/11299/253957.
