# Supplementary material for: Microfluidic investigation of the impacts of flow fluctuations on the development of Pseudomonas putida biofilms
Source: NPJ Biofilms Microbiomes. 2023 Oct 3;9:73. doi: 10.1038/s41522-023-00442-z (PMC10547774; doi:10.1038/s41522-023-00442-z)
Supplement: Supplementary file 1 — Supplementary information [file 41522_2023_442_MOESM1_ESM.pdf]

Supplementary Information

Microfluidic investigation of the impacts of flow fluctuations on the development of *Pseudomonas putida* biofilms

Guanju Wei [1, 2], Judy Q. Yang [1, 2*]

[1] Saint Anthony Falls Laboratory, University of Minnesota, Minneapolis MN 55414, USA

[2] Department of Civil, Environmental, and Geo-Engineering, University of Minnesota, Minneapolis MN 55455, USA

Corresponding Author

[*]Judy Q. Yang (judyyang@umn.edu)

Contents

Supplementary Methods 1-5

Supplementary Figures 1-12

Supplementary Video 1

Supplementary References

Notes: The x, y, z axes for all the figures denote the flow direction, lateral direction, and vertical direction, respectively.

Supplementary Method 1. COMSOL simulation

To calculate the wall shear stress, we conducted computational fluid dynamics (CFD) simulations using COMSOL Multiphysics 5.5 to simulate the flow in the microfluidic channel. The geometry of the microfluidic channel was set the same as our experimental setup. The Navier-Stokes equation was numerically solved to determine the flow velocity profiles inside the channel, with the assumption of no-slip boundary conditions on all solid boundaries. The stationary simulation was conducted in the fluid phase. Fully developed flow was assumed at the inflow and zero pressure was used at the outflow. Shear rate and shear stress distributions were calculated based on the velocity profiles in COMSOL.

Supplementary Method 2. Cell density

To further understand the fluctuating effects on biofilm growth, we calculated the cell density, which is defined here as the number of cells per square millimeter (cell number/mm^2). First, we selected a region of interest (ROI) and the area of the ROI is calculated in Image-J. Second, we measured the biofilm areal coverage in the ROI using the same approach outlined in the Methods section. Then, we divided the area of the biofilms by the area of a single cell $A_c = 3$ μm^2 (the area is approximated as half the surface of a cylinder with radius 1 μm and height 2 μm for *P. putida*) to calculate the number of the cells. To estimate the cell density, we divided the number of cells by the area of the ROI to give cells number per area (Fabbri et al., 2017).

Supplementary Method 3. Timescales

The diffusion timescales of oxygen and nutrient (D-glucose) into the microfluidic channel were estimated to be 0.9 and 3.2 seconds, respectively, where the diffusion timescales are defined as $t_{\mathrm{D}} = \frac{\xi^2}{2D}$, where ξ = 60 μm is the channel width, D is the diffusion coefficient of oxygen/glucose into water. The longest replenishment timescale for the new nutrient to replace the channel was calculated to be 7.2 second, where the advection timescale is defined as $t_{\mathrm{U}} = \frac{V}{Q}$, where V = 0.12 μL is the channel volume, Q = 1 μL/min is the lowest flow rate. Therefore, by comparing these timescales with the minimum fluctuation period of 5.6 minutes, we can infer that the availability of oxygen and nutrient inside the channel remains unaffected by the fluctuating flow conditions.

Supplementary Method 4. Derivation of the adjustment time T_0

The proposed modified biofilm growth model assumed that the biofilms do not grow during intervals of high shear and grow at a constant rate u_0 after adjustment time T_0 during low shear stress intervals (Figure 3-(a)). The adjustment time T_0 is an important parameter considered here, and we used the data from the high-frequency experiments to calculate it:

$$L_{\mathrm{Bavg\text{-}HF}} = n(T_{\mathrm{HF}} - T_0)u_0 \qquad \text{(S1)}$$

where $L_{\mathrm{Bavg\text{-}HF}}$ = 6.4 μm is the average biofilm thickness during the fluctuation phase (Figure 2-(a), here we used the mean value calculated from three replicates). T_{HF} = 5.6 minutes is the time duration of each low shear stress interval of the high-frequency fluctuating flow. T_0 is the adjustment time for bacteria to adjust to the flow and form biofilms. u_0 = 3.2 μm/hour is the growth rate of the biofilm thickness obtained by linearly fitting of biofilm thickness versus

growth time during the low shear stress interval of the low-frequency fluctuation flow (18 to 24 hours) (Supplementary Figure 8). n = 48 is the number of low-high shear stress cycles during the exponential growth period.

Supplementary Method 5. Three-dimensional visualization of biofilm distribution

To visualize the spatial distribution of biofilms in three dimensions, we stained the nucleic acids of the biofilms for four selected replicate experiments (steady flow and fluctuating flow with low, medium and high frequencies). Specifically, we used a mixture of 10 μL of SYTO-9 green fluorescent nucleic acid stain (5 μM, Thermo Fisher Scientific, USA) and 10 mL of carbon-free M9 solution as the stain solution. Following 24 hours of biofilm development experiments, we initiated the staining process by switching the valve to continuously introduce the stain mixture into the microfluidic channel at a flow rate of 0.5 μL/min for 1 hour. After that, biofilms over the channel depth were scanned at several vertical positions at 0.5 μm vertical resolution using the Z-Stack function of the Nikon NIS-Elements software. The scanning process required approximately 2 hours to be completed.

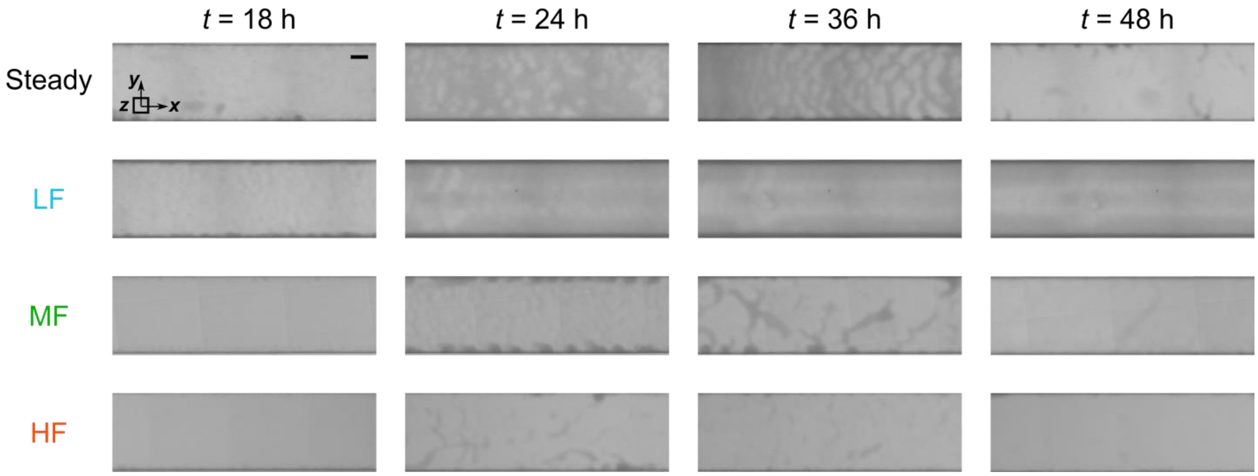

Supplementary Figure 1. Time sequence images of biofilm growth under steady flow and fluctuating flow conditions: steady-flow (frequency f = 0 Hz), low-frequency fluctuating flow (LF, $f = 2\times10^{-5}$ Hz), medium-frequency fluctuating flow (MF, $f = 2\times10^{-4}$ Hz), and high-frequency fluctuating flow (HF, $f = 1\times10^{-3}$ Hz). The scale bar on the top left image is 100 µm, and all insets have the same scale bar.

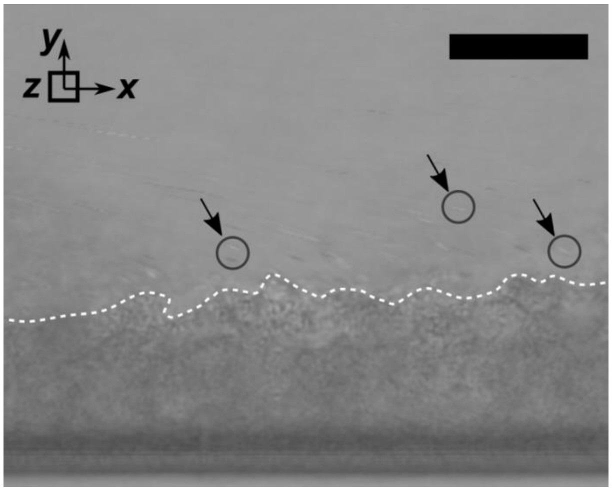

Supplementary Figure 2. Microscopy images of planktonic cells in the microfluidic channel during low shear stress. The planktonic cells exist outside the biofilms. The white dashed curve denotes the boundary of biofilms. The black arrows and circles indicate the planktonic cells. The flow condition here is low-frequency fluctuating flow. The scale bar is 25 μm.

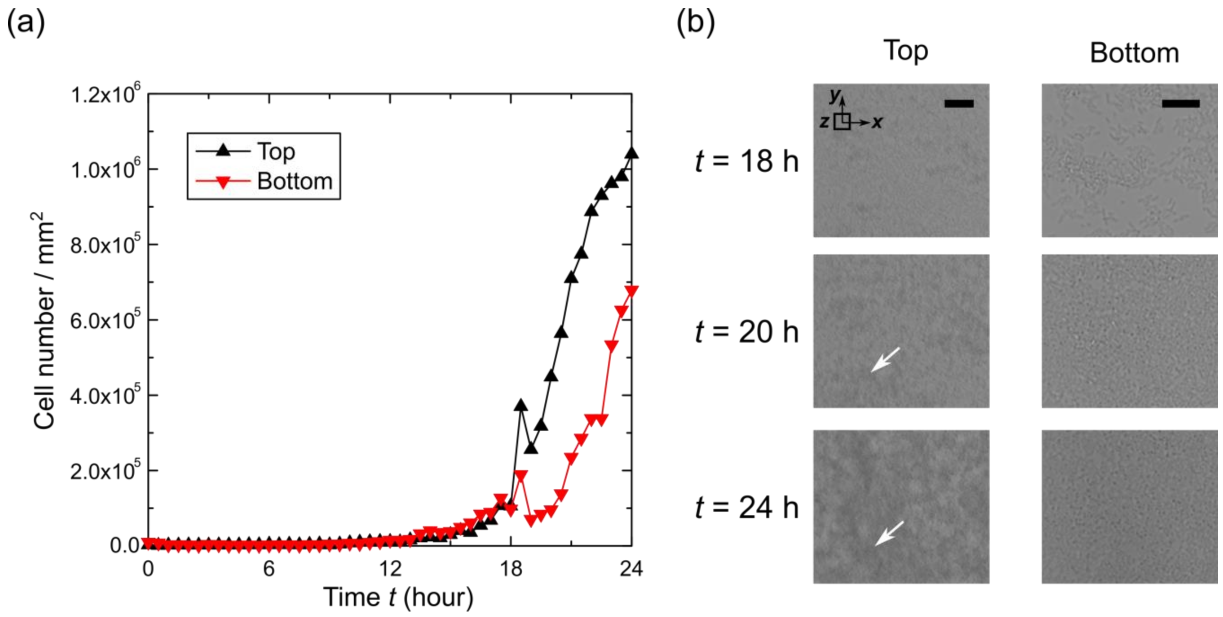

Supplementary Figure 3. (a) Cell density, defined as the number of cells per area, on the top PDMS and bottom glass surfaces during the initial 24 hours before biofilm formation. The flow condition here is low-frequency fluctuating flow. The curves represent the average values of three replicate experiments. (b) Microscopic images of bacterial cells at selected times. The white arrow highlights the initiation of biofilm formation on the top surface, while no biofilm was observed on the bottom surface. The scale bar is 10 μm.

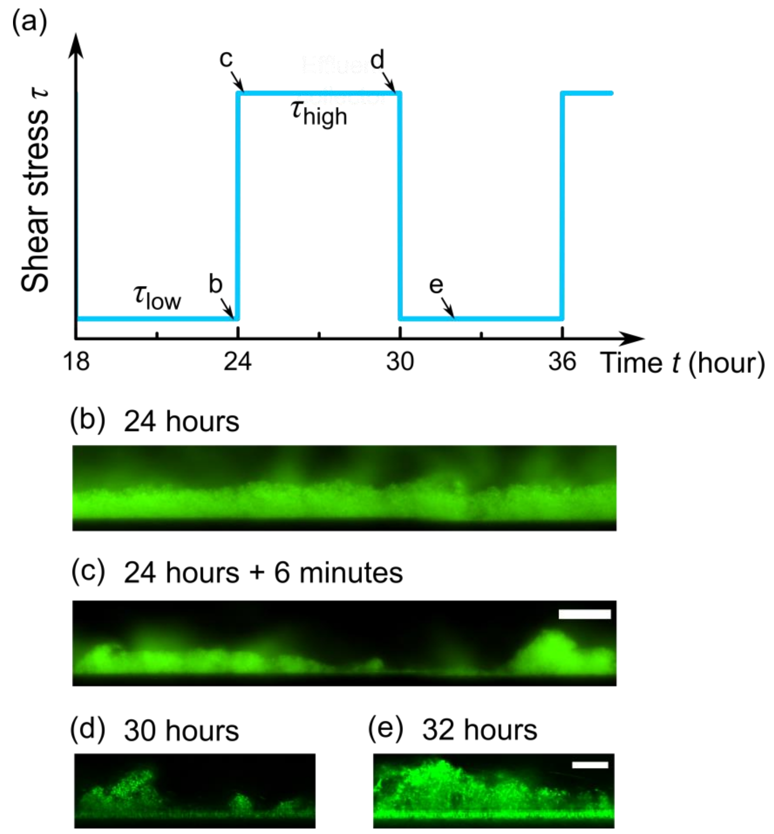

Supplementary Figure 4. Local biofilm detachment and regrowth observed by a confocal microscope. The biofilms (green color) were stained by a SYTO-9 dye. (a) Temporal profile of shear stress for the low-frequency condition (frequency $f = 2\times10^{-5}$ Hz, the duration for low or high shear stress T_{LF} = 6 hours). Images (b) and (c) shows the detachment of biofilms when the flow switches from the low shear stress (labeled as b in panel (a)) to high shear stress (labeled as c in panel (a)). The scale bar is 50 μm for both panel (b) and (c). Images (d) and (e) show the regrowth of biofilms when the flow switches from high shear stress (labeled as d in panel (a)) to low shear stress (labeled as e in panel (a)). The scale bar is 20 μm for both panel (d) and (e).

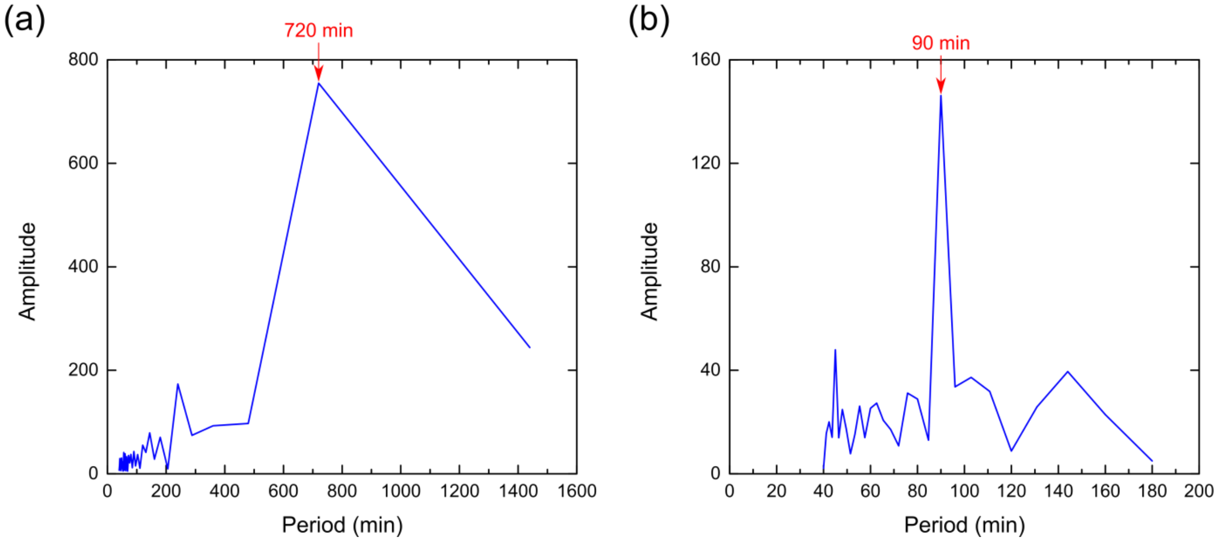

Supplementary Figure 5. Fast Fourier Transform (FFT) analysis to determine the dominant frequency/time period for (a) low-frequency (12-hour time period) and (b) medium-frequency experiments (90-minute time period).

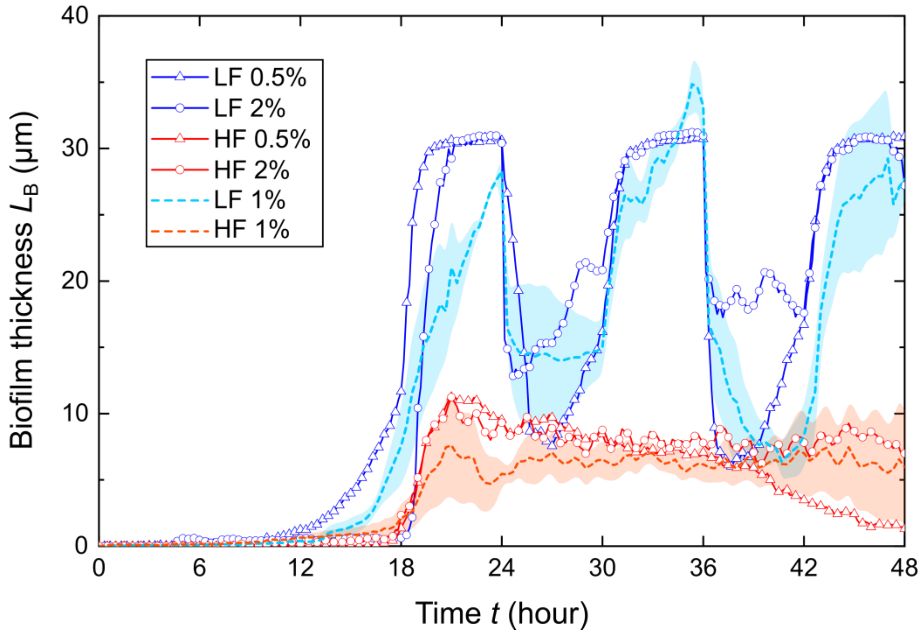

Supplementary Figure 6. Additional biofilm growth experiments were conducted with half (0.5 wt. %) and two times (2 wt. %) the current glucose concentration (1 wt. %). The dotted lines with shadows correspond to the original experimental results with standard errors observed in the experiments (the same data shown in Figure 2-(a)). The solid lines with triangle and circle represent the biofilm thickness measured at half (0.5 wt. %) and two times (2 wt. %) the original glucose concentration (1 wt. %), respectively.

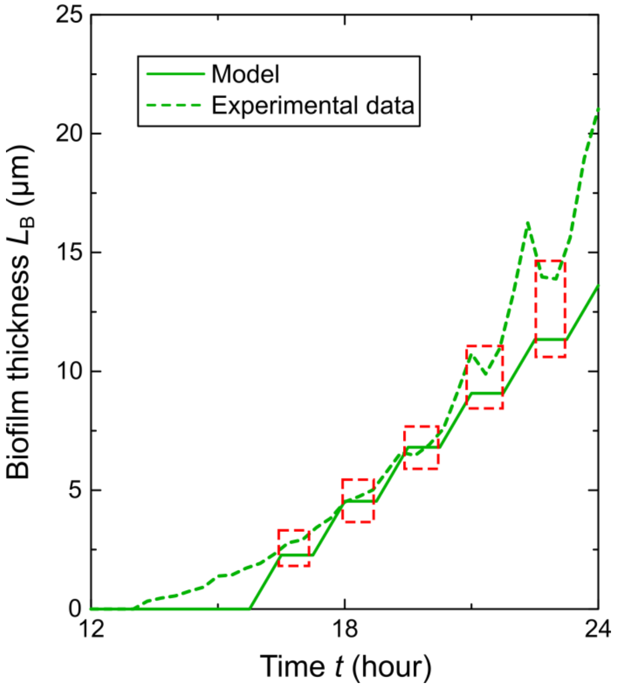

Supplementary Figure 7. Comparison between the proposed growth model (solid lines) and the measured biofilm thickness (dashed lines) during the exponential phase under medium-frequency conditions (data obtained from Figure 3-(d)). The red dashed box indicates the biofilm thickness remains relatively constant during high shear stress, consistent with the assumption of our model (Equation (2)).

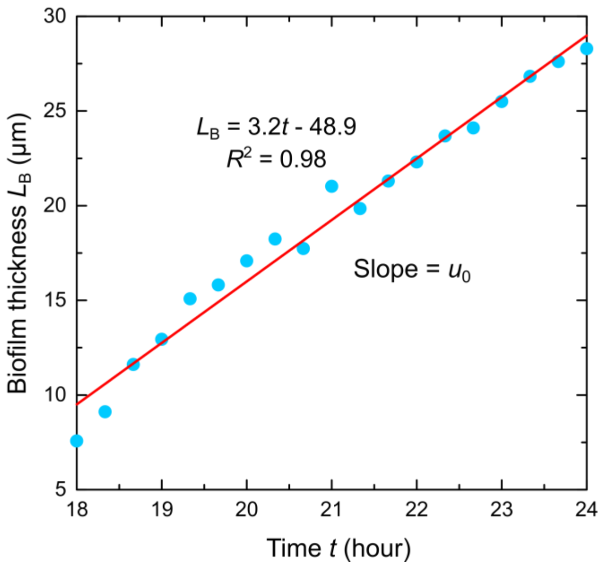

Supplementary Figure 8. Linear fit of biofilm thickness versus growth time measurements during the low shear stress interval of the low-frequency fluctuation flow (18 to 24 hours) (data obtained from Figure 2-(a)). The slope of the fit represented the constant grow rate u_0 used in the theoretical model. Here we used the mean value from three replicates.

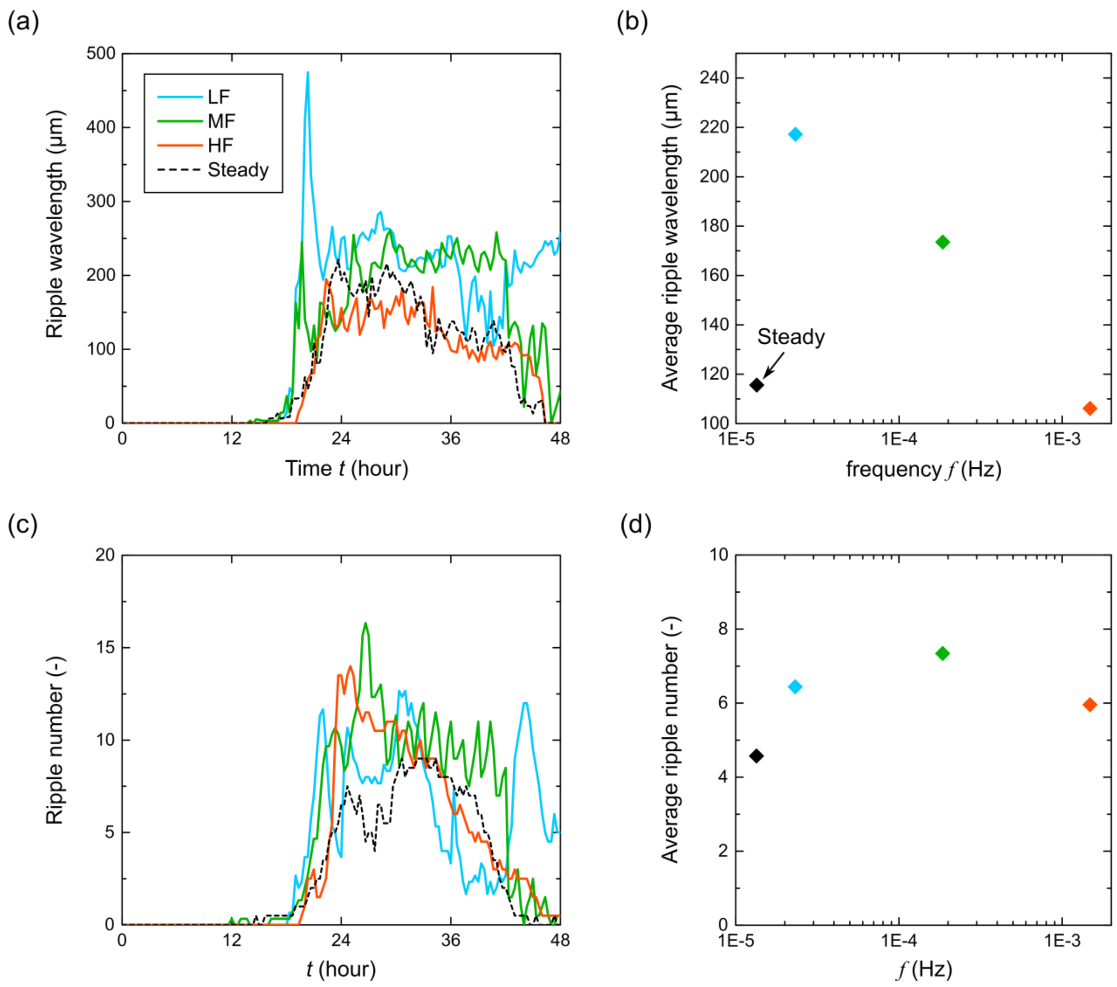

Supplementary Figure 9. Analysis of ripple parameters in response to flow fluctuations. (a) Ripple wavelength (defined as the distance between the centers of two adjacent ripple-like biofilms) and (c) ripple number (defined as the number of ripple in the field of view) as a function of time for steady flow and fluctuating flow conditions. (b) Average ripple wavelength and (d) average ripple number after 18 hours under steady flow and fluctuating flow conditions. The data represents averages of three replicates.

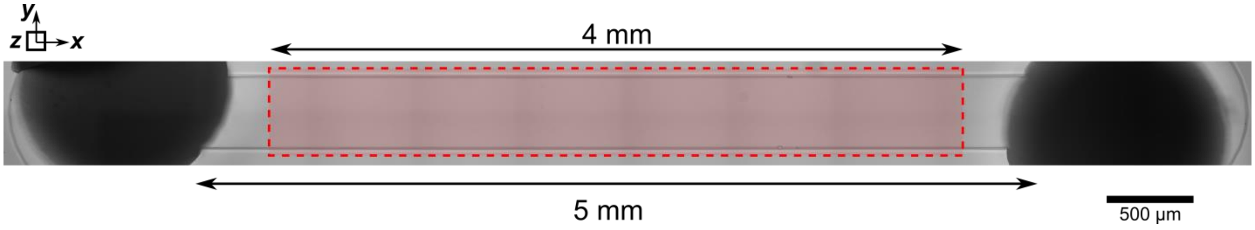

Supplementary Figure 10. Field of view for microfluidic experiments. The microfluidic channel has a length of 5 mm, extending from the inlet to the outlet. The central segment, measuring 4 mm in length and positioned 0.5 mm away from both the inlet and outlet, was used for imaging and analysis. The channel width is 400 µm. The darker regions at the inlet and outlet result from shadowing caused by the insert tubes. The scale bar is 500 µm.

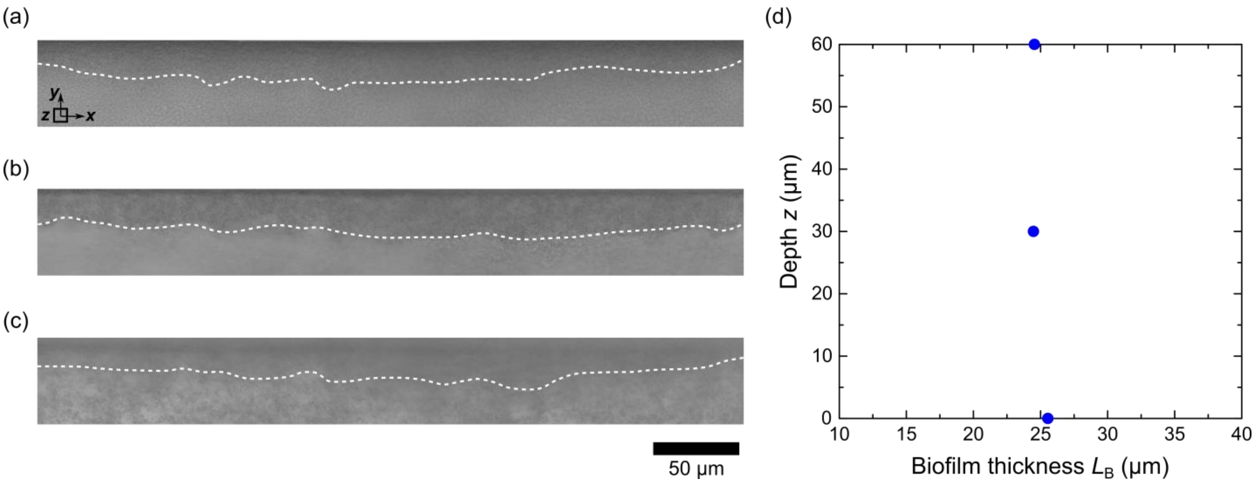

Supplementary Figure 11. Microscopic images of the biofilm on the sidewalls at different depths. Images taken at (a) the top surface (depth z = 60 µm), (b) the middle-depth plane (z = 30 µm), and (c) the bottom surface (z = 0 µm). (d) Biofilm thickness was measured at 24.5 µm (top surfaces, z = 60 µm), 24.4 µm (middle-depth plane, z = 30 µm), and 25.5 µm (bottom surface, z = 0 µm), respectively, with a mean value of 24.8 ± 0.4 µm. The scale bar is 50 µm.

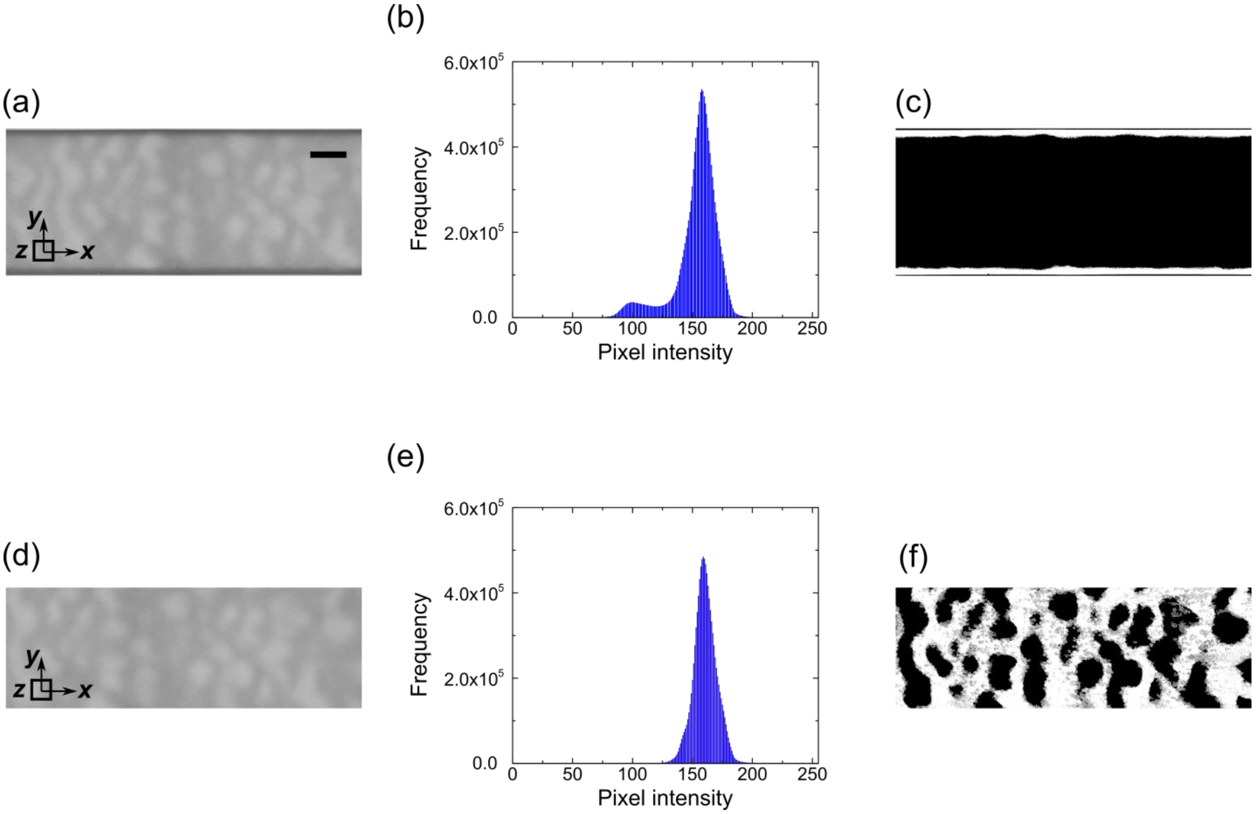

Supplementary Figure 12. Image analysis. (a) The raw images obtained from Nikon confocal microscope. The images show both the membrane-like sidewall biofilms and ripple-like biofilms on the top PDMS surfaces. (b) The two-peak pixel intensity value distribution of the images shown in panel (a). (c) The binary image using Otsu's method (Otsu, 1979). (d) The raw images obtained from Nikon confocal microscope show the ripple-like biofilms. (e) The one-peak pixel intensity value distribution of the images shown in panel (d). (f) The binary image was obtained by a manually determined threshold method, which was based on comparing the pixel intensity between the light intensity of the ripple-like biofilms and the background using Image-J. The scale bar is 100 μm for all panels.

Supplementary Video

Supplementary Video 1. Time-lapse video showing the biofilm detachment happens during the shear stress transition from low to high (after 24 hours). The flow direction is from left to right. Fluorescence images were taken every 5 seconds.

Supplementary References

Fabbri, S., Li, J., Howlin, R. P., Rmaile, A., Gottenbos, B., De Jager, M., Starke, E. M., Aspiras, M., Ward, M. T., & Cogan, N. G. Fluid - driven interfacial instabilities and turbulence in bacterial biofilms. *Environmental Microbiology*. **19**: 4417-4431 (2017).

Otsu, N. A threshold selection method from gray-level histograms. *IEEE transactions on systems, man, and cybernetics*. **9**: 62-66 (1979).
